# WHY MEASUREMENT MATTERS IN UNDERSTANDING SOCIAL CONNECTIONS: REFLECTIONS ON MEASURING SOCIAL ISOLATION

**DOI:** 10.1093/geroni/igae098.0221

**Published:** 2024-12-31

**Authors:** Mary Louise Pomeroy, Fereshteh Mehrabi, Emerald Jenkins, Roger O’Sullivan, Jim Lubben, Thomas Cudjoe

**Affiliations:** Johns Hopkins University, Baltimore, Maryland, United States; Concordia University, Montreal, Quebec, Canada; Johns Hopkins University, Baltimore, Maryland, United States; Ageing Research And Development Division, Institute Of Public Health, Dublin, Dublin, Ireland; Boston College, Chestnut Hill, Massachusetts, United States; Johns Hopkins University School of Medicine, Baltimore, Maryland, United States

## Abstract

Social isolation has a negative impact on society and increases the risk of morbidity and mortality. Despite recent strides in this literature, methodological issues remain in how social isolation is measured and subsequently described. Fundamentally, most measures are not validated, do not measure objective social isolation, or interchange assessments of social isolation with loneliness. Many studies continue to rely on a single item to measure social isolation, most likely resulting in biased estimates. Researchers are faced with a variety of unvalidated metrics that differ in their theoretical framing and composition. We believe that this heterogeneity stems in part from the lack of psychometric testing, impeding the advancement of work in this area. The lack of consistency perpetuates misunderstandings in population-based estimates or other rigorous characterizations of social isolation, precludes the comparison of findings across studies, and undercuts our understanding of intervention effectiveness. We call for the research community to employ psychometric testing of social isolation measures and to solicit input from a wide array of stakeholders to substantiate a proposed measure. This information could then be used to generate broad support for use of evidence-based measures of social isolation, promoting consistency and uptake in clinical, research, and community settings. Establishing acceptability and validity of social isolation measures is critical to developing screening and prevention strategies, designing effective interventions, and adopting practice and policies that are relevant to stakeholders.

